# Thermochemical Humidity Detection in Harsh or Non-Steady Environments

**DOI:** 10.3390/s17061196

**Published:** 2017-05-24

**Authors:** Devon Bridgeman, Francis Tsow, Xiaojun Xian, Qinan Chang, Yongming Liu, Erica Forzani

**Affiliations:** 1 Center for Bioelectronics and Biosensors, Biodesign Institute, Arizona State University, Tempe, AZ 85287, USA; dbridgem@asu.edu (D.B.); frant@earthlink.net (F.T.); Xiaojun.Xian@asu.edu (X.X.); 2 Fulton School of Engineering Matter, Transport, and Energy, Arizona State University, Tempe, AZ 85287, USA; qchang3@asu.edu (Q.C.); Yongming.Liu@asu.edu (Y.L.)

**Keywords:** sensor, humidity, detection, thermal, infrared, thermography, thermochemical, harsh, rapid, heat

## Abstract

We present a new method of chemical quantification utilizing thermal analysis for the detection of relative humidity. By measuring the temperature change of a hydrophilically-modified temperature sensing element vs. a hydrophobically-modified reference element, the total heat from chemical interactions in the sensing element can be measured and used to calculate a change in relative humidity. We have probed the concept by assuming constant temperature streams, and having constant reference humidity (~0% in this case). The concept has been probed with the two methods presented here: (1) a thermistor-based method and (2) a thermographic method. For the first method, a hydrophilically-modified thermistor was used, and a detection range of 0–75% relative humidity was demonstrated. For the second method, a hydrophilically-modified disposable surface (sensing element) and thermal camera were used, and thermal signatures for different relative humidity were demonstrated. These new methods offer opportunities in either chemically harsh environments or in rapidly changing environments. For sensing humidity in a chemically harsh environment, a hydrophilically-modified thermistor can provide a sensing method, eliminating the exposure of metallic contacts, which can be easily corroded by the environment. On the other hand, the thermographic method can be applied with a disposable non-contact sensing element, which is a low-cost upkeep option in environments where damage or fouling is inevitable. In addition, for environments that are rapidly changing, the thermographic method could potentially provide a very rapid humidity measurement as the chemical interactions are rapid and their changes are easily quantified.

## 1. Introduction

Although humidity sensing is a well-established field with a range of sensors available at low-cost, high accuracy, or other special applications, humidity sensing in harsh or complex environments is still a challenge. Some examples of harsh environments are sewers and equipment for drying prepared foods. In both of these cases, monitoring humidity may be needed for improved outcomes. For instance, relative humidity is an important factor in predicting corrosion rates in sewers. In the United States alone, it was estimated that sewer corrosion incurs a cost of 14 billion dollars annually [1]. Work has gone into building predictive models for service life in order to avoid costly sewer collapse [2,3,4]; moreover, as seen in the work of generating predictive models, humidity is an important factor and is beneficial for predicting corrosion rates. On the other hand, it has been found that in the aggressive environment of sewers, traditional electronic humidity sensors can begin to fail in as little as a few days due to the same effects driving sewer corrosion [5]. Along this line, work has been reported for development of robust sensors capable of monitoring in this environment, which includes Optical Fiber-Based Gratings [5] and Strain-Based Fiber Optics [6]. In addition, humidity detection in food processing can present a major challenge since the environment can be nearly fully dry (~0% relative humidity), and physically challenging due to the exposure to particulates, and suspended materials [7,8,9]. These environments can be described as harsh based on either punishing pH levels or highly-fouling environments.

Another challenging environment for sensing humidity is breath. Breath humidity is important for detection of hydration, and for accurately detecting other parameters related to metabolism [10], lung function [11], and renal status [12]. Scaling breath sensing technologies to clinical and consumer levels, however, is a massive challenge due to complexities in handling breath [13,14,15,16]. Breathing involves inconsistent and complex flow patterns and sampling involves massive changes in both humidity and temperature, especially in rebreathing applications. We describe this as a non-steady environment because relevant changes in humidity are occurring on timeframes of less than 1 s.

In previous works, we have developed disposable humidity sensors based on color change detection for product monitoring [17]. In this work, we demonstrate the utility of a thermal method for humidity detection, utilizing two approaches, consisting of a surface hydrophilically tuned to produce an exo- or endothermic reaction in response to humidity:

1. A thermistor-based chemical sensor, which consists of a surface-treated thermistor, enabling an inexpensive and robust contact method with an accurate low-thermal mass thermistor starting at less than $3 in bulk and simple data-acquisition circuitry, which can be insulated from harsh environments.
2. A thermographic humidity sensor, which utilizes a thermal camera to image a simple disposable sensing element, enabling a non-contact method of transducing humidity signal. Thermal infrared sensors start at as little as $8 each in bulk and consumer thermal cameras start at as little as $250 (FLIR^®^ One) [18].

In both methods, high accuracy can be achieved when applied with a proper reference, such as an uncoated thermistor (in the method 1-thermistor-based chemical sensor) or an insulated spot on the disposable imaging element (in the method 2-thermographic humidity sensor). In both approaches, the chemical modification of the thermistor or the disposable imaging agent (sensing element) have a wide variety of sensing options available. In this work, we take advantage of heat interactions involved in the hydration of a neutral salt in the presence of humidity. With this, we demonstrate the effectiveness of thermistor-based chemical detection of humidity and the characterization and effectiveness of thermographic chemical detection of humidity. 

## 2. Materials and Methods

### 2.1. Thermistor-Based Method

**Figure 1:**
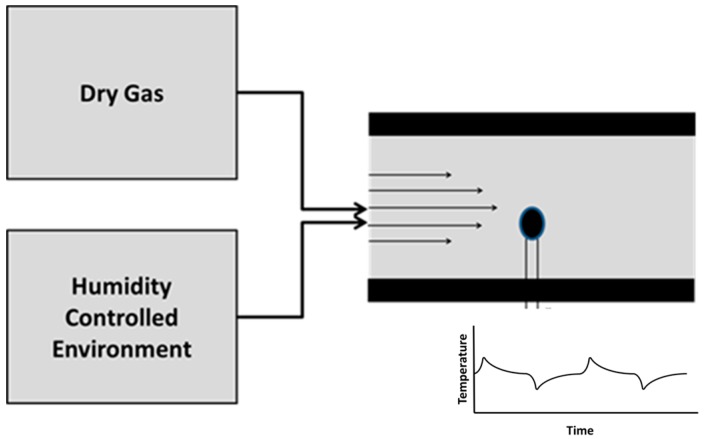
Schematic of the thermistor-based humidity detector. Alternated dry gas and humidity controlled samples were introduced at constant temperature and flow rate into the sensor chamber.

Thermistor-based method consists of a coated thermistor in a gas stream exposed to alternating samples of dry and humid gas samples (see Figure 1). For these experiments, the temperatures and flows of the two gasses were held constant, such that any heat effects were purely from water interactions. 

Sensing thermistor preparation: The sensing thermistor was prepared by coating a low-thermal mass thermistor (EPCOS, Munich, Germany, part no. B57550G0103F000) with a solution containing a long-chain quaternary ammonium salt and color dye. The salt provides heat upon hydration and the dye allows for visualization. The thermistor was dip-coated and allowed to dry in ambient lab conditions. Measurements were taken at 40 Hz, with an effective temperature resolution of less than 0.05 Celsius. 

Humidified gas samples: Humidified samples were made using a humidity controlled environment (Coy Labs). A large chamber was maintained at a humidity set point, and then gas from said environment pumped at a set flow rate over the thermistor. The flow rate was constant for all tests at roughly 3 L/min and the range of relative humidity levels probed was from 0–90% at room temperature.

### 2.2. Thermographic Method

**Figure 2:**
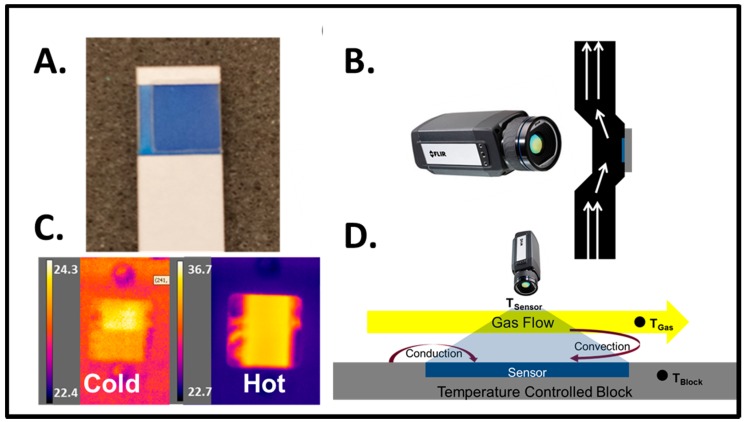
Thermographic Method: (**A**) The sensing element used for relative humidity testing. The sensor consists of a coated Teflon membrane (blue) laminated in polystyrene with cardstock for easy handling and rigidity; (**B**) An illustration of the test setup, where the camera is viewing inside of a slot in the top of the flow device, with air being passed over the sensor, and the sensor sitting in an aluminum block held at constant temperature; (**C**) A comparison between hot and cold thermal images of the setup. The warmer portion of the image is the sensor inside of the flow chamber used for the experiments, and the colder portions are the exterior of the device; (**D**) A schematic of the heat transfer phenomenon present when testing the strip. Note that relevant temperatures are measured by thermistor at the probe in the gas stream (*T_gas_*) and the sensor block (*T_block_*), along with measuring the sensor via thermal camera (*T_sensor_*).

The thermal camera setup utilized a sensor with the same chemistry as in the thermistor-based chemical method. In this case, the hydrophilic sensing mixture was applied in the form of a disposable coated Teflon strip. The Teflon strip was placed in a heated aluminum block (see Figure 2). For the thermographic tests, heat accounting was performed for general heat transfer, including conduction and convection.

Disposable sensing element: The sensing elements were prepared by coating a porous Teflon membrane with the same mixture as the thermistor. The Teflon is dip-coated and dried, then laminated into an easy-to-handle chip, as seen in Figure 2A.

Flow measurements and heating: Flow measurements and heating were performed in a custom setup. Flow measurements were performed with a differential pressure flow meter (Confined Pitot Tube [19] with a Freescale MP3V5004DP differential pressure transducer). Heat was supplied to an aluminum heating block via a Nichrome wire, with a temperature set point of 37.9 degrees Celsius. This temperature is designated as *T_block_*. The device was designed such that the sensing element (sensor) sits in the aluminum block and the flow laterally runs over the sensor. The imaging port is located in the opposite side of the sensing element, as seen in Figure 2B. An additional thermistor (same as above) was located in the flow stream to take the gas stream temperature, *T_gas_*. This temperature, together with *T_block_* and the temperature measurements assessed via the thermal camera (see below) on the sensing element (*T_sensor_*) were also taken in the gas stream in order to build the heat model used for evaluation. For these measurements, flow rates from 0–32 L/min were considered.

Thermal camera: A FLIR A655sc was used for the experiments. The camera utilizes an uncooled micro bolometer sensor array with a 640 × 480 resolution, 50 Hz full-window resolution, and accuracy of ±2% of the reading. All data acquisition was done in the provided software: Research IR.

Humidified gas samples: Samples were prepared by passing dry gas through a water bath. Samples with 100% relative humidity were prepared by passing the gas through pure water and 33% relative humidity samples were prepared by passing the gas through a saturated magnesium chloride solution.

## 3. Results and Discussion

Humidity measurements were performed using both methods: thermistor-based detection and thermographic detection, both of which were utilizing the same hydrophilic chemical probe, consisting of a mixture of a salt with a dye. With this type of chemical probe, the latent heat of vaporization of water and the enthalpy of hydration for the salt can be taken advantage of for thermal detection. With constant flow and temperature conditions, the temperature change from this reaction can be directly related to humidity change. If the flow and temperature are not constant, further analysis can be performed to relate the temperature change to humidity change by accounting for conduction and convection effects in the system. In addition, no heat release was observed with exposure to acid gasses, such as pure carbon dioxide or concentrated hydrochloric acid.

### 3.1. Thermistor-Based Humidity Detection

**Figure 3:**
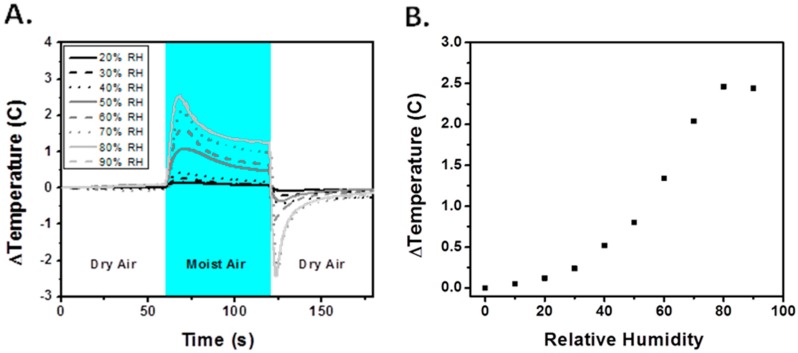
(**A**) Test results from alternating between dry and humid air. Absolute temperatures during the tests ranged from about 23 to 26 degrees Celsius; (**B**) The difference between the baseline and the peak temperature increases as a function of relative humidity.

Figure 3 shows the result of the thermistor-based humidity sensing experiments. The experiments were carried out at room temperature with insignificant fluctuations in temperature between tests as indicated by a hydrophobically (PTFE)-coated thermistor sensor used as the reference. In order to control for the effect of flow rate on the sensor, the same flow rate was used for all thermistor tests. Therefore, all temperature changes in the hydrophilically-modified thermistor were related to the humidity changes. As seen in all experiments shown in Figure 3A, there is a significant increase in temperature with humidity exposure. The temperature and flow of the stream were held constant, so heat gained is gradually lost to the stream. Due to this, we see a spike and decrease in temperature rather than accumulation. Since the temperature and flow rates were held constant, for analysis purposes, only the difference between the baseline and peak temperature from humidity exposure was needed. Figure 3B shows the relationship between the relative humidity that the sensor is exposed to and the corresponding observed temperature change. It can be seen that the thermistor-based sensor provides a proportional correlation between the temperature change and the relative humidity, and saturates around 75% relative humidity. The sensor saturation effect could be potentially combatted by either changing the chemical coating or heating the thermistor.

### 3.2. Thermal Camera Humidity Detection

The second method was to utilize a thermal camera to measure temperature changes on a disposable sensor. In order to measure heat interactions due to humidity, the sensor was held at an elevated temperature and sample gases were introduced at known flow rates. The elevated temperature works to prevent the sensor from saturation (as seen with the thermistor), which also works to protect the sensor from water accumulation. Heating was done with an aluminum block holding the sensor (see Figure 2D). Although higher temperatures are desirable for avoiding saturation, the temperature also needs to be low enough to allow for hydration. For this set of experiments, the temperature of the aluminum block holding the sensor was set at 37.9 degrees Celsius. For this set of tests, the flows and temperatures were not held constant like in the thermistor-based method. Due to this, changes in inlet temperature and flow rate needed to be accounted for.

The sensing system utilizes heat generation from the hydration of the hydrophilic chemical layer on the sensing element for the quantification of humidity levels. In order to relate the amount of heat generated from humidity interactions, the system needed to be characterized in terms of heat contributions from conduction and convection. Therefore, heat contributions from the three heat sources: conduction, convection, and chemistry were accounted for. Equation (1) shows the overall heat relationship:

(1) $$\frac{dQ_{total}}{dt}=\frac{dQ_{conduction}}{dt}+\frac{dQ_{convection}}{dt}+\frac{dQ_{chemical}}{dt}$$

where, *Q* is heat flux, either total or from a contributor (conduction, convection, or chemical interaction) as a differential over time (*t*). Equation (2) shows the relationship between the total heat and the mass (*m*), specific heat capacity (*c*), and the temperature of the sensor (*T_sensor_*):

(2) $$Q_{total}=m\times c\times \Delta T_{Sensor}=m\times c\times T_{Sensor}$$

By utilizing a reference state of 0 degrees Celsius, we can simplify Equation (2) to what is seen on the right side of the equation. The relationship can be further broken down to Equation (3) as follows:

(3) $$\frac{m\times c}{A}\times \frac{dT_{Sensor}}{dt}=K\times \left(T_{Surface}-T_{Sensor}\right)+H\left(f\right)\times \left(T_{Gas}-T_{sensor}\right)+B\times \frac{dC}{dt}$$

where, *A* is the area, *K* is the conduction coefficient, *T_surface_* is the temperature for the block, *T_sensor_* is the temperature of the sensor, *H*(*f*) is the convection coefficient as a function of the flow rate (*f*), *B* is the amount of heat generated per unit change in relative humidity concentration, *C* is the concentration of humidity on the sensor, and *t* is time. This form gives explicit contributions from each component in terms of coefficients, which were determined under certain experimental conditions as described in the next sections.

### 3.3. Heat Transfer Characterization of the Thermographic Sensing System

#### 3.3.1. Conductive Heat Transfer Characterization

The first characterization of the system was to perform heat tests without a flow in order to determine the conduction and free convection rates. In order to measure changes under these conditions, a sensor at room temperature was placed inside of the heated device, and then the rate of heating recorded via thermal camera. These tests were performed at constant relative humidity with no flow. Under these conditions Equation (3) can be simplified to Equation (4).

(4) $$\frac{m\times c}{A}\times \frac{dT_{Sensor}}{dt}=K\times \left(T_{Surface}-T_{Sensor}\right)+H\left(0\right)\times \left(T_{Gas}-T_{sensor}\right)$$

At steady-state conditions, the net heat flux will be equal to zero, which allows for simplification down to Equation (5).

(5) $$\frac{K}{H\left(0\right)}=\frac{\left(T_{sensor}-T_{Gas}\right)}{\left(T_{Surface}-T_{Sensor}\right)}$$

**Figure 4:**
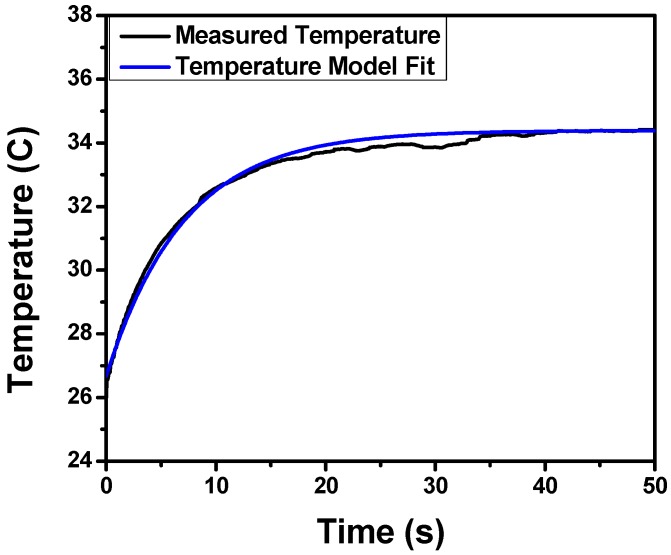
A non-flow test showing the change in temperature of the sensor over time after the room-temperature sensor is inserted into the heated device measured via thermal camera.

In order to determine the absolute values of the coefficients, the relationship can be solved for non-steady-state conditions, as seen in Figure 4. By performing this analysis, we can ascertain both *K* and *H*(0) for our system. The analysis yielded a *K* value of about 36 W/m·K and an *H*(0) value of about 24 W/m·K.

#### 3.3.2. Convective Heat Transfer Characterization

**Figure 5:**
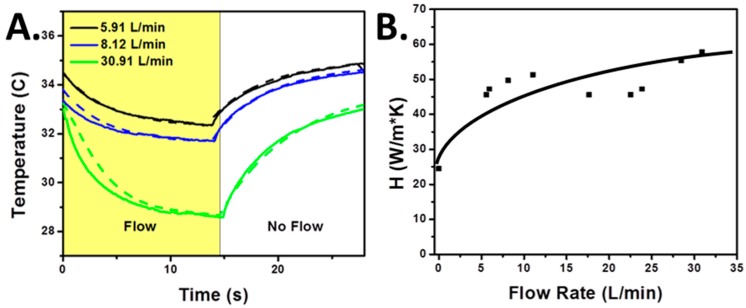
(**A**) Curves demonstrating the testing (solid line) and fitting (dotted line) procedure used to generate the heat transfer convention coefficients (*H*). Flows were pulsed and the changes in temperature were recorded and modeled. The fitting (dotted line) was performed following Equation (4); (**B**) Corresponding convection coefficient of the sensor (*H*) as a function of the volumetric flow rate.

The next step was to determine the relationship of the convection coefficient as a function of flow rate. This was done by setting a dry gas cylinder to a set flow rate and cycling flow through the setup (on and off), all while recording the temperature changes with the thermal camera. This allows a very straight forward and simple method for assessing the convection coefficient at constant flows, as all temperatures and the conduction coefficient are accounted for in all cases. Figure 5A shows a series of these tests. From these tests, the convection coefficient (*H*) as a function of the flow rate can be obtained, as shown in Figure 5B. Note that the behavior at low flows is not characteristic of a typical flat-plate cooling relationship where a fit as shown in Figure 5B would be seen [20]. This is due to the unique geometry of the system, but for simplicity we can approximate the trend with the fit seen.

#### 3.3.3. Chemical Interaction Characterization

For probing humidity with this setup, the tests can be reframed as the deviation from heat values expected under dry conditions. The heat production will be a function of two factors: the partial pressure of water in the gas and sensor, and the absorption and desorption rates, as shown in Equation (6) as follows:

(6) $${\left(\frac{m\times c}{A}\times \frac{dT_{Sensor}}{dt}\right)}_{Reaction}=B\times \frac{dC}{dt}=B\times (r_{abs}\times \left[C_{Gas}\right]-r_{des}\times \left[C_{Sensor}\right])$$

where, *r*_abs_ and *r*_des_ are the rates of absorption and desorption, respectively, *C_gas_* is the concentration of analyte in the gas surrounding the sensor, and *C_sensor_* is the concentration of analyte in the sensing element, where in this case the analyte is humidity.

**Figure 6:**
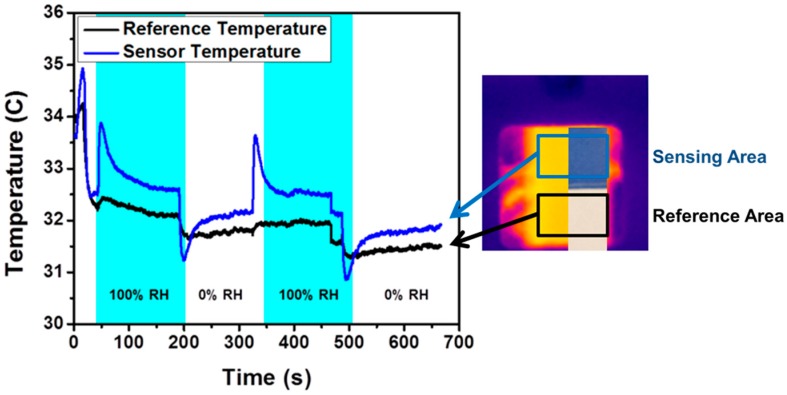
A humidity test with temperature measurements from the chemically coated sensing area and inert reference area. The reference area was a polystyrene portion of the sensor next to the sensing area.

Since the effects of conduction and convection can be calculated, the heat generation from reactivity can be ascertained. Figure 6 demonstrates that the bulk of the changes seen in the sensor temperature are in fact from humidity changes. The figure contrasts the differences seen between an uncoated reference (black line) and a coated sensor (blue line). Comparing these two, the temperature change due to humidity is seen clearly in the coated sensor.

**Figure 7:**
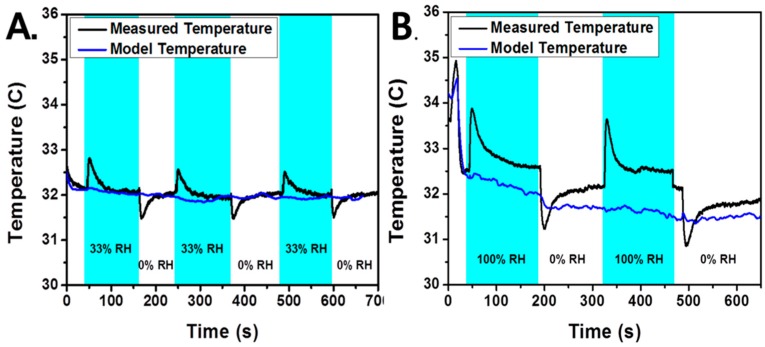
Temperature traces which show the measured heat response from pulsing alternating relative humidity levels over the sensor with dry air and air at 33% relative humidity (**A**) and 100% relative humidity (**B**). The blue line represents expected temperature values based on conduction ad convection relationships, while the black line represents the measured temperature on the hydrophilic area.

Figure 7 shows that when applying different concentration of relative humidity to the disposable sensor, we can see distinct changes in temperature with the thermal camera by averaging the appropriate areas. Clear differences in magnitude can be seen between 33% relative humidity and 100% relative humidity. Using the appropriate model, it is feasible to measure relative humidity at a fast capture rate (<1 s): the heat from humidity can be isolated from other conductive and convective effects, and the heat flux is a direct function of the change in water content of the sensor. 

## 4. Conclusions

In this work, we demonstrate the utility of the humidity detection via thermal analysis. This can provide a robust and inexpensive measurement for application in harsh environments, such as sewers or food processing operations, or in otherwise difficult environments, such as breath. Using a coated thermistor, we demonstrate the ability to discriminate relative humidity in the range of 0–75% by measuring temperature changes. Using a thermal camera and disposable element, we demonstrate a clear signal due to relative humidity change and provide evidence of a relationship between the magnitude of change in relative humidity and the magnitude of the signal. Future work in this area will be directed to specific sensor design to adapt the needs of different sensing scenarios.

## References

[1] Koch G.H., Brongers M.P., Thomson N., Virmanio Y., Payer J.H., Kutz M. (2005). Cost of corrosion in the united states. Handbook of Environmental Degradation of Materials.

[2] Jiang G., Keller J., Bond P.L., Yuan Z. (2016). Predicting concrete corrosion of sewers using artificial neural network. Water Res..

[3] Wells T., Melchers R. (2016). Life-Cycle of engineering systems: Emphasis on sustainable civil frastructure. Proceedings of the Fifth International Symposium on Life-Cycle Civil Engineering (IALCCE 2016).

[4] Alwis L.S., Bustamante H., Bremer K., Roth B., Sun T., Grattan K.T. A pilot study: Evaluation of sensor system design for optical fibre humidity sensors subjected to aggressive air sewer environment. Proceedings of the 2016 IEEE SENSORS.

[5] Alwis L.S., Bustamante H., Bremer K., Roth B., Sun T., Grattan K. (2016). Evaluation of the durability and performance of FBG-based sensors for monitoring moisture in an aggressive gaseous waste sewer environment. J. Lightwave Technol..

[6] Thomas P.J., Hellevang J.O. (2017). A fully distributed fibre optic sensor for relative humidity measurements. Sens. Actuators B Chem..

[7] Sebastian P., Bruneau D., Collignan A., Rivier M. (2005). Drying and smoking of meat: Heat and mass transfer modeling and experimental analysis. J. Food Eng..

[8] Zhang W., Ma H., Yang S.X. (2016). An inexpensive, stable, and accurate relative humidity measurement method for challenging environments. Sensors.

[9] Nathakaranakule A., Kraiwanichkul W., Soponronnarit S. (2007). Comparative study of different combined superheated-steam drying techniques for chicken meat. J. Food Eng..

[10] Zhao D., Xian X., Terrera M., Krishnan R., Miller D., Bridgeman D., Tao K., Zhang L., Tsow F., Forzani E.S. (2014). A pocket-sized metabolic analyzer for assessment of resting energy expenditure. Clin. Nutr..

[11] Bridgeman D., Zhao D., Tsow F., Xian X., Forzani E. A non-invasive and inexpensive capnography device for the monitoring of copd and other pulmonary diseases. Proceedings of the IEEE Healthcare Innovation Point-of-Care Technologies Conference.

[12] Davies S., Spanel P., Smith D. (1997). Quantitative analysis of ammonia on the breath of patients in end-stage renal failure. Kidney Int..

[13] Qian Z. (2016). The Impact of Humidity on an Optical Chemical Sensing Device for Non-Invasive Exhaled Gas Monitoring. Master’s Thesis.

[14] Jalal A.H., Umasankar Y., Gonzalez P.J., Alfonso A., Bhansali S. (2017). Multimodal technique to eliminate humidity interference for specific detection of ethanol. Biosens. Bioelectron..

[15] Zito C.A., Perfecto T.M., Volanti D.P. (2017). Impact of reduced graphene oxide on the ethanol sensing performance of hollow SnO_2_ nanoparticles under humid atmosphere. Sens. Actuators B Chem..

[16] Yao M.S., Tang W.X., Wang G.E., Nath B., Xu G. (2016). Mof thin film-coated metal oxide nanowire array: Significantly improved chemiresistor sensor performance. Adv. Mater..

[17] Bridgeman D., Corral J., Quach A., Xian X., Forzani E. (2014). Colorimetric humidity sensor based on liquid composite materials for the monitoring of food and pharmaceuticals. Langmuir.

[18] FLIR. http://www.flir.com/home/.

[19] Bridgeman D. The development of a new differential pressure flow meter for bidirectional measurement of human breath flow. Proceedings of the American Institute for Chemical Engineers 2015 Annual Meeting.

[20] Whitaker S. (1972). Forced convection heat transfer correlations for flow in pipes, past flat plates, single cylinders, single spheres, and for flow in packed beds and tube bundles. AIChE J..

